# Talking Mats and self-perceived involvement for service recipients with dementia in Swedish home care services: a randomized controlled trial

**DOI:** 10.1186/s12877-026-07518-3

**Published:** 2026-04-28

**Authors:** Angela Bångsbo, Anna Dunér, Tina M. Olsson

**Affiliations:** 1 Department of Work Life and Social Welfare, University of Borås, Borås, Sweden; 2 Department of Social Work, University of Gothenburg, Gothenburg, Sweden; 3 Department of Social Work, Jönköping University, Jönköping, Sweden

**Keywords:** Talking Mats, Dementia, Home care services, Involvement, Satisfaction, Randomized controlled trial

## Abstract

**Background:**

Older people with dementia are regularly excluded from decision-making processes and their wishes and preferences for care are often not respected. The aim of this study was to analyze whether the use of Talking Mats^®^ (TM) help older service recipients with mild to moderate dementia to feel more involved in and satisfied with needs assessment and planning conversations within Swedish home care services, compared to usual conversation methods.

**Methods:**

This study employed a post-test only, two-armed parallel group randomized controlled trial design. The use of TM was evaluated in comparison with a control group using usual conversation methods. Inclusion criteria were service recipients from participating municipalities, ≥ 65 years scoring 12-23p on the MMSE. Randomization was conducted in blocks by site *(n* = 3) with allocation set at 50% per arm with concealed allocation. Once participants were randomized to study arm blinding was not possible. Post-tests were conducted with service recipients after conversations with care managers and nursing assistants. The impact of staff’s perceived acceptability, appropriateness and feasibility of TM was assessed.

**Results:**

101 older home care services recipients were recruited, 51 in the TM group and 50 in the control group. Non-response was relatively high > 10% in both groups. Total attrition showed 24% of the service recipients with dementia did not complete data collection following conversations with CMs, and 35% with NAs. High involvement and satisfaction were found in both TM and control group in conversations with care managers and nurse assistants. No difference between groups was detected. Dementia severity was significantly and positively correlated with self-perceived involvement.

**Conclusions:**

There were no significant differences between TM and control groups regarding self-perceived involvement and satisfaction after conversations with care managers and nurse assistants.

**Trial registration:**

ClinicalTrials.gov ID: NCT05 561,998. Registered on September 28, 2022.

## Introduction

Globally, dementia is one of the major causes of disability and dependency among older people. In 2021, 57 million people worldwide lived with dementia, and 10 million new cases are estimated each year [1]. Yet, people with dementia are regularily excluded from decision-making processes and their wishes and preferences for care are often not respected [2]. Thus, there is a need to develop and evaluate communication tools in dementia care, to enable service recipients with dementia continued opportunities of influence and control.

In Sweden, the number of people living with dementia is estimated to be approximately 160,000 and in those over 90 years of age, approximately 50% live with dementia [3]. 72% of Swedes with dementia were living in ordinary housing, among whom 50% were receiving home care services [4]. If an older person can no longer manage independently, he or she can apply for assistance from the local eldercare authority. A care manager (CM) performs a needs-assessment, and support may include help with household chores, personal care, and/or social activities. The Swedish Social Services Act states that eldercare should aim at strengthening older people’s ability to live an independent life, in dignity, and with well-being [5, 6]. Developments in Swedish eldercare have, during last decades, been dominated by a drive for individualized support, with an emphasis on consumer choice [6, 7], the intention being more customized services for the individual as well as increased quality of the services provided. In the Swedish National guidelines on dementia care [3], it is stressed that people living with dementia are covered by the same policy intentions as other groups using eldercare. In some other countries, like the Netherlands and England, decision-making support for older people making choices about social care have been developed [8, 9]. However, Sweden lacks such support even though research have shown that it may enable older people living with dementia to communicate their needs and preferences with CMs and nursing assistants (NAs) in home care services [10–12]. Thus, we initiated a study to evaluate the use of Talking Mats^®^ (TM), a picture-based low-technology communication tool, within the normal operating of eldercare services of three municipalities in southwestern Sweden. TM were tested in conversations between service recipients with mild to moderate dementia and CMs and NAs when assessing needs and planning delivery of home care services, compared with usual conversation methods (UCM) in the same context.

### Previous research

Several studies conducted within the context of Swedish home care services show that situations involving needs-assessment and provision of home care services for older people with dementia often are very challenging [10–13]. Most CMs lack training in communicating with people with dementia and they face several dilemmas in relation to the exchange of information as well as regarding conflicting interests between various actors involved in the assessment of support needs for people with dementia [11, 13–15]. In addition, NAs who perform care and services express difficulties with interpreting the wishes of older people with dementia [12, 13]. Still, it has been shown that CMs’ behaviour in conversations with older people living with dementia can both facilitate or restrain self-determination [16–18]. Instead of being involved, people with dementia are more often talked about than talked to and are thus excluded from decisions about their future care [19–21]. To allow service users with dementia continued opportunities for influence and participation, methods to support decision-making may be required [9, 22–24].

Two systematic reviews of decision supports for older adults show that decision aids had the potential to increase knowledge, create more accurate risk perceptions, and help participants choose options more congruent with their values [25, 26]. Such decision supports were often designed as visual aids intended as conversation props. In previous research, the picture-based communication tool TM has shown promising results in facilitating the communication, involvement and choice, and decision-making of people with communication difficulties, even though the level of research evidence is limited [27, 28]. For people with dementia, TM can support them to feel more involved in making choices and decisions about things that matter to them [13, 28–30]. Additionally, TM may improve their ability to understand topics by providing a visual cue which allows more time to process information [13, 30]. The TM framework may provide a tool that staff can use to engage and help people with dementia express their views about a range of topics and thus play an important role in improving their quality of care [31].

TMs could be used by people at both early, middle, and late stages of dementia. However, communication was improved in particular for people with early and moderate stages of dementia [32, 33]. In one study, the use of TMs was also associated with better communication for people with more advanced stages of dementia [34]. Even though previous research has shown that decision support would be welcomed by CMs and NAs in Swedish eldercare [11, 12], the use of such support when communicating with older people with dementia is very limited. To our knowledge, no previous studies have studied the use of TM as a communication tool and decision aid in the context of Swedish home care services.

### Aims and research questions

The aim of this study was to analyze whether the use of Talking Mats^®^ (TM) help older service recipients with mild to moderate dementia to feel more involved in and satisfied with needs assessment and planning conversations within Swedish home care services, compared to the use of UCM, as well as to analyze the impact of participants’ dementia severity and home care staff’s perceived acceptability, appropriateness and feasibility of TM on outcomes.

Specific research questions are:

- RQ1. To what extent does the use of TM impact service recipients’ self-perceived involvement in and satisfaction with conversations about home care services with CMs and NAs, compared to the use of UCM?
- RQ2: To what extent does dementia severity impact self-perceived involvement and satisfaction in those conversations?
- RQ3: Are there differences in CMs’ and NAs’ perception of the acceptability, feasibility, and appropriateness of the TM method directly following training compared to after using TM with at least one service recipient with mild to moderate dementia?
- RQ4: To what extent does the CMs’ and NAs’ perception of the acceptability, appropriateness and feasibility of TM after at least one use impact service recipients’ self-perceived involvement in and satisfaction with conversations about home care services with CMs and NAs?

## Methods

### Study design

The complete study design have previously been described in a study protocol [35]. In this article we address one aim and RQ 1 described in the study protocol by analysing data on the primary outcome measure of the study, perceived involvement and satisfaction of older participants with dementia. In order to deepen our understanding of how well TM works we have added additional RQs and developed the analysis by using background data on dementia severity of participants and data on participating staffs’ perceptions of acceptability, appropriateness, and feasibility of TM. In forthcoming publications we will analyse recorded conversations between service providers and service recipients with dementia as well as qualitative focus group data on the implementation.

We employed a post-test only, two-armed parallel group randomized controlled trial design. The use of TM, the intervention, was evaluated in comparison with a control group using UCM during 2022–2024. Participants in the intervention group used TM as a decision aid in needs assessment conversations as well as in conversations about the delivery of home care services. Post-tests were conducted with older service recipients with mild to moderate dementia after conversations with two groups of service providers (T1) CMs and (T2) NAs. Data on older participants’ involvement and satisfaction was collected directly following needs-assessment conversations reviewing decisions about home care services with municipal CMs (T1) and directly following conversations with NAs about planning of delivery of home care services chosen during the needs assessment (T2). In addition, data on participating staffs’ perceptions of acceptability, appropriateness, and feasibility of TM was collected twice. They all received an online questionnaire, first after TM training (T*a*) and secondly after having at least one TM conversation (T*b*).

### Setting and procedure

The study was conducted within the normal operating of eldercare services of three municipalities in southwestern Sweden, a detailed description of participating municipalities can be found in the study protocol [35]. This meant that a pragmatic approach was required, i.e. to balance sound research methodology with what was practically feasible in the study setting. For such reasons, the participating CMs and NAs conducted the conversations with both the intervention TM group and the control group participants. Moreover, high staff turnover meant that new staff had to be recruited throughout the study period, and only a few staff participated long enough to be truly experienced TM users. A further challenge was the declining health and energy of older service recipients with dementia, affecting their opportunities to participate in the study. This made the process of recruiting participants more difficult and led to a relatively high rate of non-response. Therefore, the planned qualitative interviews were deemed as too demanding to complete. Continuous meetings and workshops were held between researchers and managers, and research assistants throughout the study period, to discuss the design and conduct of the study, address any upcoming issues, and monitor participant enrolment and adherence to study protocol. Furthermore, home care service practitioners, policy makers, and user representatives were invited to discuss the planning, implementation and findings of the study in seminars arranged by the local Research & Development unit ’FoU Välfärd’ and the National Dementia Association.

CMs and NAs were recruited through eldercare managers in the three municipalities based on their interest to take part in the study. Service recipients with mild to moderate dementia were identified by CMs, NAs or professionals from a specialized dementia team. Potential participants received a brief information about the study and were offered the opportunity to participate in screening tests for mild to moderate dementia. It was made clear that the choice to participate in the study (or not) did not affect receipt of eldercare. After expression of interest to participate and permission to forward contact details to the researchers, service recipients were contacted by a research assistant.

To collect data from older service recipients with dementia, three visits were conducted at each participant’s home. At the first visit, participants were screened to ensure that they met the inclusion criteria. The included participants were randomized into intervention group to use TM in conversations with both CMs and NAs or control group using UCM in conversations with both CMs and NAs. Within one month the second visit took place where the needs assessment was conducted by a CM. At the third visit, conducted within seven weeks after screening, a NA performed the planning of delivery of home care services.

Due to the challenges of ensuring informed consent from older people with dementia, participants were informed about the project on several occasions. Initially, they received both oral and written information from using visual clues and plain language together with a formal, more comprehensive information sheet that was also given to their next of kin. Oral and written informed consent were collected before data collection started. Thereafter, the participants’ oral consent was requested in connection with each data collection occasion.

### Participants

#### Older service recipients with mild to moderate dementia

101 older home care services recipients were included in the study, 51 in the TM intervention group and 50 in the control group. Inclusion criterias for participants were aged ≥ 65 years with mild to moderate dementia (Mini-Mental State Examination (MMSE, PAR Inc. Psychological Assessment Resources) score 12–23) and receiving home care services in one of three participating municipalities. Participants with severe vision loss were excluded.

Participant *(n* = 101) characteristics of the service recipients can be found in Table 1. Participants were 59% (*n* = 60) female and 41% (*n* = 41) male with a mean age of 84.2 (*SD* = 6.7) years. The majority of the sample (*n* = 93; 92%) scored between 12 and 23 on the MMSE (total range 10–26). Participants mean years in education was 8.99 years (*SD* = 2.90). We conducted comparisons between the TM and UCM groups on attrition and found no differences in total attrition across groups and no differences based on background characteristics (sex, age, MMSE, educational attainment, dementia severity, balance, and gait speed).

**Table 1:** Participant characteristics of home care services recipients (*n* = 101) in TM (*n* = 51) and UCM (*n* = 50) groups

Variable	Total sample *n* (%)	TM group *n* (%)	UCM *n* (%)	X^2^ (*p*)
Sex				0.87 (0.35)
Female	60 (59)	28 (55)	32 (64)	
Male	41 (41)	23 (45)	18 (36)	
Age (M, sd, t)	84.18 (6.69)	84.43 (6.32)	83.92 (7.11)	(0.82)
Education	8.99 (2.90)	9.38 (2.94)	8.60 (2.83)	(0.19)
MMSE	18.69 (3.60)	18.18 (4.05)	19.22 (3.03)	(0.15)
Berg balance scale	37.04 (10.52)	36.41 (10.94)	37.61 (10.20)	(0.60)
Gait speed	0.50 (0.18)	0.49 (0.18)	0.52 (0.19)	(0.40)

*M* mean, *sd* standard deviation, *t* independent samples t-test, *p* p-value

The significance level is 0.05

Age, MMSE were tested with Mann-Whitney U

Education, Berg balance scale, Gait speed were tested with independent samples t-test

#### Home care staff

A total of 67 home care staff (e.g., leadership, program developers, CMs and NAs) participated in the completion of the study. Of which, 18 cm and 14 NAs were engaged in the delivery of the TM and UCM conversations. Inclusion criteria for staff participants were employment in one of the three municipalities and voluntary interest to participate in the study. Among the home care staff, CMs had university degrees in social work or other social sciences, and most of the NAs had completed upper-secondary education as assistant nurses. All but two home care staff were female, and their ages varied between 22 and 53 years. Their professional experience ranged from one to 31 years.

### Interventions

#### Talking Mats (TM)

**Fig. 1:**
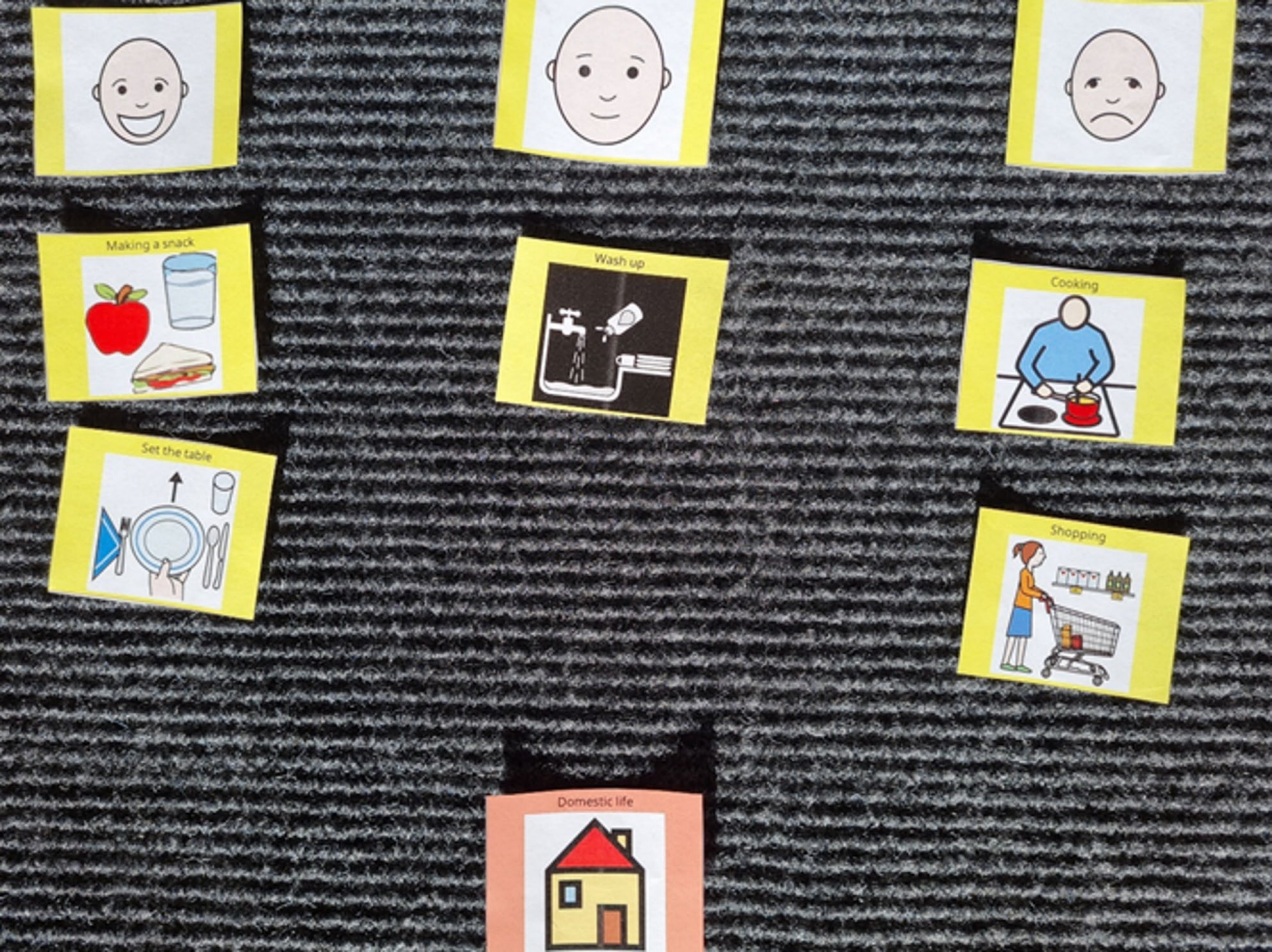
TM example with topic, a visual scale, and a set of options

TM framework was developed and evaluated at the University of Stirling, Scotland [26, 30]. TM consists of a range of topics and options. In this study, the topics and options were developed from the World Health Organization’s (WHO) International Classification of Functioning [36] to represent domains of activity and participation. Researchers, CMs, and NAs collaborated to develop files with picture symbols (see example, Fig. 1) in color which were available from a free online picture resource [37]. The picture symbols illustrate activities such as self-care (dressing, eating), domestic life (cooking, shopping), and participation (e.g., interpersonal interactions, relationships). More specifically, the picture symbols were chosen to support the needs assessments and planning of home care services. All participating CMs and NAs received a two-days TM training, after which they were licensed to use TM.

#### Usual Conversation Methods (UCM)

UCM represents the regular conversation method used by CMs and NAs in their conversations with older service recipients with dementia. The CMs’ needs-assessment conversations are based on the systematic needs-assessment tool IBIC [38].

### Measures

#### Background and demographic measures

Data was collected on participants’ sex (male, female), age, education (years of school completed), and cognitive and physical functioning.

##### Mini-Mental State Examination (MMSE)

The MMSE is an 11-item measure that serves as a contribution to diagnose dementia, testing cognitive function: orientation, registration, attention and calculation, recall, language, comprehension, and ability to read and write. The maximum score is 30 and a score of 23 or lower is indicative of cognitive impairment [39–41].

##### Berg Balance Scale (BBS)

The BBS determines an individual’s ability to balance through observation of 14 predetermined tasks, each graded on a 5-point ordinal scale indicating functional levels from low (0) to high [4] with 56 as maximum total score. Score of < 45 indicates a greater risk of falling [42].

##### Gait speed test (4-meter)

A gait speed of < 0.8 m/s on a four-meters walking distance indicates an increased risk of frailty [43].

#### Primary outcome measure

##### Self-perceived Involvement

We used the Involvement Measurement Scale (IMS) to assess participants’ self-perceived involvement with decision making related to home care services. The IMS consists of six questions. Questions 1–5 are measured on a 4-point Likert scale ranging from “never/none” to “always/all”. The IMS is taken from the Freedom of Choice Interview Schedule [44] which is a measure specifically designed for people with dementia and their family carers and measures respondents’ self-perceived involvement in care issues. We have adapted and translated to Swedish [45] the version used by Murphy et al. [29] for this study [35].

##### Perceived satisfaction

Question six from the IMS is specifically designed to measure participant satisfaction. Satisfaction is measured on a 7-point Likert scale ranging from “not very well at all” to “very well indeed”.

The IMS was administered by the researchers directly following the conversations with CMs (T1) and NAs (T2). Each question and response alternative were read out and were sometimes explained to ensure the service recipient could complete the questionnaire.

#### Perceived acceptability, appropriateness, and feasibility

An online questionnaire was sent twice to assess the participating staffs’ perceptions of acceptability, appropriateness, and feasibility of TM. At the initial survey (T*a*), the leadership, internal program developers, care managers, and nurse assistants (*n* = 67) who had finished at least one-day of TM training received the questionnaire. The questionnaires were administered directly following the training during 2020–2023. A second questionnaire (T*b*, *n* = 32) was sent during 2024 to CMs (*n* = 18) and NAs (*n* = 14) who had used TM at least once after attending the training. Their experience of using TM varied from 1 to 10 conversations. A total of 47 (70%) of participating managers, internal program developers, CMs, and NAs completed the questionnaire following training, and 25 (78%) of CMs and NAs completed the questionnaire after using TM. We used the following measures including a response scale from “completely disagree” to “completely agree”.

##### Acceptability of Intervention Measure (AIM)

Perceived acceptability of TM is measured with 4 items on a 5-point Likert scale [46].

##### Interventions Appropriateness Measure (IAM)

Perceived appropriateness of TM is measured with 4 items on a 5-point Likert scale [46].

##### Feasibility of Intervention Measure (FIM)

Perceived feasibility of TM is measured with 4 items on a 5-point Likert scale [46].

### Sample size

We conducted an a priori sample size calculation. To detect a moderate difference (0.25 effect size) with 0.9 power, and a p-value set to 0.05, a minimum sample size of 50 participants was determined to be needed [42]. Prior studies of TM have found significant differences in self-perceived involvement and satisfaction measured with the IMS after using TM and in comparison to UCM in much smaller samples (i.e., *n* = 18) [29].

#### Randomization and blinding

**Fig. 2:**
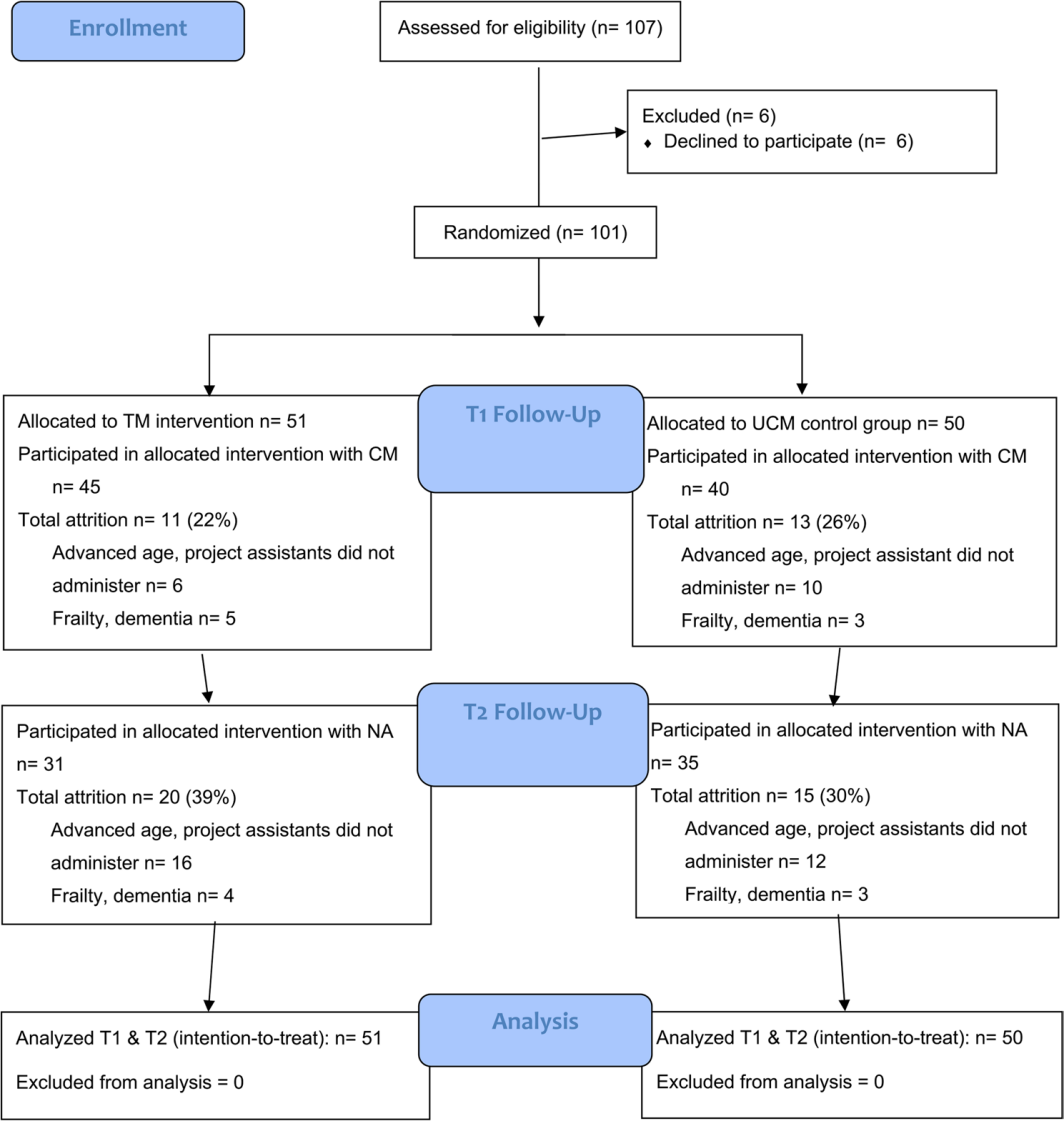
Flow chart of participating service recipients with mild to moderate dementia

The randomization schedule was generated using an online random number generator (random.org). The randomization was conducted in blocks by site *(n* = 3) with allocation set at 50% per arm. The randomization schedule was unknown to participating municipalities or members of the research team involved in collaboration activities and data collection. Results of the randomization were communicated only after study inclusion and the collection of baseline measurement data. To ensure allocation concealment, randomization was conducted by a non-operational researcher for each included participant. Once participants were randomized to study arm, blinding was not possible (Fig. 2).

### Statistical analyses

IBM SPSS Statistics for Windows, version 31.0 (IBM Corp., Armonk, NY), was used for all statistical analyses. Characteristics of participating service recipients are reported with descriptive statistics (e.g. percent, mean) depending on variable measurement characteristics. Differences between participants in each study arm at baseline are tested with Chi^2^ or the standard independent means t-test depending on variable characteristics. Differences between groups on the primary outcome were assessed with the standard independent means t-test and Mann-Whitney U test. Depending on data characteristics, we used multiple linear regression and the non-parametric Spearman’s rho to assess the relationship between participant characteristics and provider perceptions on outcome. As 7% (*n* = 8) of the sample fell outside of the range set for assessment of mild to moderate dementia, we conducted sensitivity analyses using the reduced sample of *n* = 93 to test the robustness of our results. This did not produce changes in primary results and therefore we use the entire sample in the analyses presented here.

#### Intent-to-treat

We conducted an intent-to-treat (ITT) analysis using multiple imputation and pooled results across imputed data sets. Total attrition showed that 24% (22% TM; 26% UCM) of the service recipients with dementia did not complete data collection following conversations with CMs and 35% (39% TM; 30% UCM) did not complete data collection following conversations with NAs.

## Results

### Impact of TM compared to the use of UCM on service recipients’ self-perceived involvement and satisfaction (RQ1)

There were no significant differences between TM and UCM groups in self-perceived involvement or satisfaction following conversations with CMs or NAs (Table 2).

**Table 2:** T-test results self-perceived involvement and satisfaction after CMs and NAs conversations (*n* = 101)

	TM Mean (SE) *n* = 51	UCM Mean (SE) *n* = 50	t (*p*)	95% CI Diff.
T1 (CMs)				
Self-perceived involvement	3.82 (0.06)	3.62 (0.08)	0.52 (0.60)	− 0.14 − 0.24
Satisfaction	5.07 (0.167)	5.18 (0.16)	0.46 (0.65)	− 0.36 − 0.57
T2 (NAs)				
Self-perceived involvement	3.88 (0.07)	3.64 (0.07)	1.00 (0.32)	− 0.80 − 0.26
Satisfaction	5.31 (0.152)	5.45 (0.16)	0.54 (0.59)	− 0.31 − 0.53

*t* independent samples t-test

### The impact of dementia severity on outcome (RQ2)

Dementia severity was significantly and positively correlated with service recipients’ self-perceived involvement in conversations at T1(*r* = .382, *p* = .002) and T2 (*r* = .287, *p* = .009). Dementia severity was not significantly correlated with satisfaction at T1 (*rho =* 0.168, *p* = .13) or T2 (*rho =* 0.093, *p* = .45).

### Differences in staffs’ perceived acceptability, appropriateness, and feasibility of Talking Mats directly following training compared to after using TM (RQ3)

Home care staffs’ perceptions of acceptability (T*a* = 4.52, T*b* = 3.95, *p* *≤* .001), appropriateness (T*a* = 4.13, T*b* = 3.37, *p* *≤* .001), and feasibility (T*a* = 4.05, T*b* = 3.70, *p* = .02) of using TM was significantly higher for home care staff surveyed directly following training compared to staff surveyed after experience using TM in conversations with service recipients.

### The impact of staffs’ perceived acceptability, appropriateness, and feasibility on service recipients’ self-perceived involvement and satisfaction (RQ4)

Service recipients’ self-perceived involvement in conversations within the TM group at T1 (*M* = 3.82, *SE* = 0.06) was moderately correlated with self-perceived involvement at T2 (*M* = 3.88, *SE* = 0.07; *rho* = 0.54, *p* = .05). When controlling for self-perceived involvement at T*a*, perceived acceptability did not significantly impact self-perceived involvement at T*b* (R^2^Δ = 0.002, *F*Δ (1, 82) = 0.259, *p* = .65). Similarly, perceived appropriateness did not significantly impact self-perceived involvement at T*b* when controlling for self-perceived involvement at T*a* (R^2^Δ = 0.001, *F*Δ (1, 82) = 0.126, *p* = .76). This result was similar for perceived feasibility (R^2^Δ = 0.002, *F*Δ (1, 82) = 0.235, *p* = .67) which did not significantly impact self-perceived involvement at T*b* when controlling for self-perceived involvement at T*a*.

Perceived satisfaction within the TM group at T*a* (*M* = 5.07, *SE* = 0.167) was moderately correlated with satisfaction at T2 (*M* = 5.31, *SE* = 0.152; *rho* = 0.387, *p* = .03). Perceived satisfaction at T*a* was not correlated with perceived acceptability (*rho* = 0.003, *p* = .98), perceived appropriateness (*rho* = − 0.05, *p* = .75), or perceived feasibility (*rho* = 0.003, *p* = .98). Similarly, perceived satisfaction at T*b* was not correlated with perceived acceptability (*rho* = 0.075, *p* = .69), perceived appropriateness (*rho* = 0.153, *p* = .41), or perceived feasibility (*rho* = 0.08, *p* = .69).

## Discussion

In this study, we analyzed whether the use of TM may help older home care service recipients with mild to moderate dementia to feel more involved in and satisfied with needs assessment and planning conversations within the context of Swedish home care services. The results showed no significant impact of the use of TMs on older service recipients self-perceived involvement or satisfaction following conversations with CMs or NAs. Thus, this result differs from many previous studies pointing to positive results of Talking Mats regarding several communication aspects and involvement in conversations for people with various communication difficulties [28–30]. However, most previous research on TM has been conducted in small-scale, descriptive studies and no previous randomized controlled trials have been conducted [27].

Further, the results showed that all participants with dementia displayed high levels of self-perceived involvement and satisfaction which limits the ability of new methods to improve on these levels [47]. However, correlational analyses revealed that self-perceived involvement within the TM group at T1 was moderately correlated with self-perceived involvement at T2. This may indicate that service recipients who are generally positive are positive across measurement points and that those who are not remain so. Thus, the results may have as much to do with individual characteristics of service recipients as with TM. Moreover, in this study, conversation partners in both the TM and UCM groups were qualified CMs or NAs, who can be assumed to have relatively good knowledge on how to communicate with service recipients with dementia. In addition, there may be reason to suppose that undergoing TM training may have improved the quality of the conversations for home care staff continuing with UCM as well.

Additionally, we analyzed to what extent dementia severity impacted self-perceived involvement and satisfaction. Dementia severity of participants was significantly and positively correlated with their self-perceived involvement following conversations with both CMs and NAs. However, dementia severity was not significantly correlated with satisfaction. Our results showed that as dementia severity increases, so does self-perceived involvement in conversation. Thus, these results agree with a study of Murphy et al. [34], indicating that the use of TMs can be associated with better communication also for people with more advanced stages of dementia. Most previous TM studies have showed positive results in particular for people with early and moderate stages of dementia [32, 33]. As dementia progresses, individuals typically experience a decline in cognitive function, leading to reduced ability to engage in decision-making and planning. Thus, with increased dementia, Talking Mats may be useful to improve perceived involvement in conversations around home care services.

Furthermore, we examined the differences in perceived acceptability, appropriateness and feasibility as perceived by home care staff following training and after using TM with at least one service recipient with mild to moderate dementia. Participating staff perceived the acceptability, appropriateness, and feasibility of using TM as significantly higher when surveyed directly following training compared to following having used TM in conversations with study participants. A qualitative focus group study on the views of the CMs and NAs on the implementation of Talking Mats showed that they considered TM conversations as qualitatively better and helpful to older people living with dementia to be more involved [13]. However, CMs regarded it unrealistic to use TM for entire needs assessment conversations. Moreover, CMs and NAs saw TM conversations as more time consuming than UCM, and described that conversation participants were “stuck” in a seated position unable to move about. Similarly, results from another study showed that TM discussion was experienced as less spontaneous, more time-consuming, and needing more preparation than conversations without TM [48]. Thus, the survey results presented in our study suggest that even though CMs and NAs embrace the idea of using TM, the more practical experience they get, the more shortcomings they may encounter.

Finally, we analyzed to what extent the CMs and NAs perceived the acceptability, appropriateness and feasibility of TM after using TM in conversations with older service recipients in relation to service recipients’ self-perceived involvement and satisfaction. As already mentioned, self-perceived involvement in TM group at T1 was moderately correlated with self-perceived involvement at T2. However, home care staff’s perceptions on TM did not impact the self-perceived involvement of participants with mild to moderate dementia. Possibly, this may emphasize how the professionals strive to empower, be objective, and avoid influencing choice and decision-making [49].

### Method discussion

The current study represents the first randomized controlled trial of TM and the first study of TM conducted within elder care in Sweden. In addition, this study uses a clear and objective measure of mild to moderate dementia, used outcome measures which have been used in prior studies of TM [29, 34], and followed implementation by assessing providers’ perceived acceptability, appropriateness and feasibility of TM with widely used and validated instruments. An additional strength of this study is that it was conducted within the normal operations of elder care services in Sweden, which adds to the study’s external validity and the overall ability to generalize its results within the Swedish elder care context. Yet despite these strengths, some limitations should be discussed.

For pragmatic reasons, the CMs and NAs who implemented the TM conversations in this study also conducted the conversations with control group participants. As such, the TM training may also have influenced their delivery of UCM, and thus we cannot ignore the risk that contamination may have impacted study results.

Moreover, this study had a relatively high rate of non-response (> 10%) in both groups caused by advanced age, project assistants lost to administer the questionnaire, dementia and frailty. As such, the study may suffer from attrition bias. However, in our attrition analyses, we found no indication that total attrition differed between TM and UCM groups. Similarly, we did not find any differences in attrition among subgroups of participants, which increases our confidence that attrition was equally dispersed across groups.

Because of staff turnover, sickness, or parental leave, etc., the group of home care staff surveyed directly following TM training (T*a*) and those surveyed after experience using TM in conversations with study participants (T*b*) are not made up of entirely the same group of individuals. As such, changes in perceived acceptability, appropriateness and feasibility over time could not be assessed in the current study. A related issue is that we were unable to link individual provider perspectives directly with individual TM participant outcomes. This would have aided in our understanding of the relationship between provider perceptions and TM participant outcomes and should be attempted in future research in the field.

Furthermore, prior research has raised questions regarding the presence of a ceiling effect and concerns regarding lack of clear cut-off scores associated with the implementation measures used in this study [50]. Consistent with prior research, TM providers rated their perceived appropriateness, acceptability, and feasibility as high on the Likert scales used. If a ceiling effect does exist, this would limit our ability to detect a true difference between participants and therefore lead to inaccurate conclusions regarding provider perception and the relationship of these perspectives with TM participant outcomes.

Also, a certain amount of heterogeneity in our studied population is evident as 7% of participants fell outside of our stated range for mild to moderate dementia [35]. These participants were screened and included due to communication failure in the beginning of the study. Although we ran analyses on the full and reduced samples and found no differences between groups, the reduced sample analyses are somewhat underpowered. In addition, given the relatively high scores on self-perceived involvement, we cannot rule out a ceiling effect on this measure [51]. Moreover, non-parametric tests were conducted for ordinal data measures when para-metric analysis where not adequate. However, the tests showed similar results.

Finally, this study was conducted as a post-test only randomized trial. This design was appropriate for our research question(s) and was also deemed relevant to our study population. The design does, however, place limits on our ability to assess and control for pre-intervention differences. That said, our pre-test analyses give no indication that the randomization procedure did not work as expected as we found no differences across groups on pre-test measures. This increases our confidence that even unmeasured differences were equally distributed across our intervention groups in line with the randomized design.

## Conclusions

Conversations supported by TM within Swedish home care services for service users with mild to moderate dementia displayed high levels of self-perceived involvement and satisfaction. But there was no significant difference between TM intervention group and the UCM control group. Participants’ dementia severity was significantly and positively correlated with their self-perceived involvement in needs assessment and planning conversations with home care staff. However, further studies are needed to examine in which stages of dementia it is most helpful to introduce the TM framework.

## Data Availability

The datasets used during the current study are available from the corresponding author on reasonable request.

## Abbreviations

TM: Talking Mats^®^
UCM: Usual Conversation Methods
CM: Care Managers
NA: Nursing Assistants
